# The Use of Nanomaterials in Tissue Engineering for Cartilage Regeneration; Current Approaches and Future Perspectives

**DOI:** 10.3390/ijms21020536

**Published:** 2020-01-14

**Authors:** Aziz Eftekhari, Solmaz Maleki Dizaj, Simin Sharifi, Sara Salatin, Yalda Rahbar Saadat, Sepideh Zununi Vahed, Mohammad Samiei, Mohammadreza Ardalan, Maryam Rameshrad, Elham Ahmadian, Magali Cucchiarini

**Affiliations:** 1 Pharmacology and Toxicology Department, Maragheh University of Medical Sciences, 5515878151 Maragheh, Iran; 2 Dental and Periodontal Research Center, Tabriz University of Medical Sciences, 5166614756 Tabriz, Iran; 3 Department of Pharmaceutical Nanotechnology, Faculty of Pharmacy, Tabriz University of Medical Science, 5166614756 Tabriz, Iran; 4 Nutrition Research Center, Tabriz University of Medical Sciences, 5166614756 Tabriz, Iran; 5 Kidney Research Center, Tabriz University of Medical Sciences, 5166614756 Tabriz, Iran; 6 Faculty of Dentistry, Tabriz University of Medical Sciences, 5166614756 Tabriz, Iran; 7 Natural Products and Medicinal Plants Research Center, North Khorasan University of Medical Sciences, 9414975516 Bojnurd, Iran; 8 Student Research Committee, Tabriz University of Medical Sciences, 5166614756 Tabriz, Iran; 9 Center of Experimental Orthopaedics, Saarland University Medical Center, D-66421 Homburg/Saar, Germany

**Keywords:** nanomaterial, cartilage tissue engineering, regenerative medicine

## Abstract

The repair and regeneration of articular cartilage represent important challenges for orthopedic investigators and surgeons worldwide due to its avascular, aneural structure, cellular arrangement, and dense extracellular structure. Although abundant efforts have been paid to provide tissue-engineered grafts, the use of therapeutically cell-based options for repairing cartilage remains unsolved in the clinic. Merging a clinical perspective with recent progress in nanotechnology can be helpful for developing efficient cartilage replacements. Nanomaterials, < 100 nm structural elements, can control different properties of materials by collecting them at nanometric sizes. The integration of nanomaterials holds promise in developing scaffolds that better simulate the extracellular matrix (ECM) environment of cartilage to enhance the interaction of scaffold with the cells and improve the functionality of the engineered-tissue construct. This technology not only can be used for the healing of focal defects but can also be used for extensive osteoarthritic degenerative alterations in the joint. In this review paper, we will emphasize the recent investigations of articular cartilage repair/regeneration via biomaterials. Also, the application of novel technologies and materials is discussed.

## 1. Introduction

Cartilage is an important tissue that exists in animal and human bodies but despite other tissues, it does have no nerves, blood vessels or lymphatics [1]. Most cartilage tissues are in charge of large mechanical loads for preparing a lubricated and smooth surface to help transmit of mechanical loads through a low friction coefficient [2]. Therefore, cartilage tissues are prone to injuries. Osteochondritis, aging, trauma, along with cancer and endocrine pathologies are the common causes of cartilage defects [3]. The healing and repair of the defected cartilage is not easily facilitated. Therefore, the repair of a normal function and structure of the impaired cartilage is a challenging issue in sports medicine and orthopedic investigations. In spite of the efforts made in this regard, the existing restoration strategies are unable to respond to the biochemical and biological characteristics of articular cartilage [4].

Tissue engineering suggests a potential approach which seeds chondrogenic cells on biocompatible scaffolds in order to produce engineered cartilage for the injured one [5]. In previous tissue engineering methods, seeding and penetration of cells were not accomplished efficiently by using rigid scaffolds [6]. Beyond rigid scaffolds, cell therapies, scaffold-free strategies and hydrogels have been reported to be promising approaches to overcome the large cartilage defects [7,8,9]. For cartilage repair, the transplantation of matrix-based autologous chondrocyte has recently been proven as a novel tactic [10,11]. The term “repair,” in tissue engineering field, can be included in two separate procedures: replacement and regeneration. Replacement is a kind of healing process whereby severely damaged or non-regenerable tissues are repaired by the laying down of connective tissue. However, in the regeneration process (as a kind of healing process), the portions of a damaged tissue completely return to their normal form [12].

Nanotechnology is defined as the science of manipulation of matter at the nanometer scale and nanomaterials describe materials that have at least one dimension in the nanometer size range. At the nanoscale level, materials behave very differently compared to larger scales and nanomaterials show unique physical and chemical properties in comparison to their bulk form [13,14,15]. Particulate nanomaterials can also be combined with biomaterials which can stimulate the native cartilage’s extracellular microenvironment. This in turn increases the cells interaction with the fabricated scaffold to progress the utility of the subsequent engineered construct [16]. Nanoscale features are believed to affect the cell behavior to a great extent, resulting in alterations in the cell shape, cytoskeleton, motility, and focal adhesions along with the expression of different genes [16].

Biomaterial tools, genetic manipulations, and cell sources have extensively grown in the last two decades, having a positive effect on the expansion of truly functional engineered-tissue [17]. Due to the present practical limitations connected to the application of cell sources and autologous adult cells in the clinic, this field has speedily turned to other progenitor cell origins [18]. Mesenchymal stem cells (MSCs) could be derived from adipose or other tissues [19]. Furthermore, the application of embryonic stem cell (ES)-derived progenitors and induced pluripotent stem cells (iPSCs) to construct cartilaginous tissues seems to be the newest strategies [20].

## 2. Articular Cartilage

The complex structure of articular cartilage needs to be understood prior to the development of a mimicking construct. The articular cartilage has the main responsibility of transmitting the loads to the related subchondral bone, then absorbing the impact forces, leading to the enhancement of a smooth, low-friction, and sliding motion of that joint [16]. When a cartilage is subjected to a persistent load, these functions compromise its rheological viscoelastic properties. Cartilage performance changes with time, declining its prominence by half of its original dimension in a lifetime and when this degeneration or trauma commences, its recovery course is too slow. Articular cartilage does not have a self-healing ability specifically due to its low chondrocyte activity and lack of vascularity [21].

The extracellular matrix (ECM), which consists of water, collagens, proteoglycans, and non-collagenous glycoproteins, forms the articular cartilage as a connective tissue. Also, cell-secreted hyaluronan, as well as serum-derived hyaluronan-related proteins which have a covalent attachment to this polysaccharide, are found in the ECM [22,23]. Collagen type II forms the majority of the dry weight of a mature cartilage, and other collagen types have small amounts which are interconnected by hyaluronan and proteoglycans, constituting about 10% of its total weight [24]. The mechanical specifications of articular cartilage (stiffness and tensile strength) are due to triple-helix structure of cartilage collagen fibers, which restrain and immobilize the negatively charged proteoglycans inside the ECM [25]. The hydrophilicity of proteoglycans leads them to be involved in cartilage compressive loading resistance, which is based on water pressurization, determining its permeability according to the proteoglycans amount [7]. Moreover, the higher stiffness and strength of tissue are caused by the globular protein domains of proteoglycans [26]. Hyaluronan is a non-sulfated glycosaminoglycan, which coats around each chondrocyte and its rheological properties offer great tensile strength to the density of cartilage tissue [27]. Further, hyaluronan is substantially involved in cellular growth and migration, and a transitory hydrated setting contributing to cell migration by assisting cellular detachment is provided by its physicochemical properties [28].

Eighty percent of the total weight of articular cartilage is due to water molecules, supporting the elasticity and lubricity as along with the action of transporting nutrients [29]. The chondrocyte is the only cell type found in the matrix, which occupies about 5%–10% of the total tissue volume [30,31]. Articular cartilage has four major zones: calcified, lower, middle, and superficial, resulting in a highly organized cell distribution in these four regions that, based on some morphological features (cell size, shape, arrangement and expression of proteoglycans, collagen and hyaluronan), can be distinguished [32]. These morphological features differ in their genotype, phenotype and functions [33]. In the superficial zone, a low number of proteoglycans exists, collagen fibers align with the surface in a parallel manner, and chondrocytes are flattened and elongated [34]. Moving towards the middle zone, the presence of proteoglycans becomes greater, the random arrangement of collagen exists, and chondrocytes become rounded [35]. Collagen fibers within the lower zone, align vertical to the bone, and the main cell category gathers itself in columns [36]. Approaching the calcified zone, more chondrocytes have a tendency to express several forms of collagen, and the production of ECM is increased [37]. Chondrocyte metabolism is affected by growth factors, electrical fields, matrix composition, mechanical loads, and hydrostatic pressures [38]. In the presence of small amounts of oxygen, an aerobic metabolism occurs. Articular chondrocytes, their metabolism, and generation of different ECM molecules regulate the regeneration and remodeling of articular cartilage [39].

## 3. Current Clinically Approaches for Cartilage Tissue Engineering

Currently applied surgical manipulations in cartilage reconstruction consist of marrow stimulation including microplasty, microfracture, and autologous chondrocyte implantation (ACI) [40]. The latter is generally utilized for extensive symptomatic defects in which a non-osteoarthritic cartilage encloses the defects [40]. For this, a permissive niche should be provided by the injured site in order to deposit new tissue via implanted cells. So, the conventional technique of chondrocyte injection under a periosteal patch has been replaced using biodegradable 3-D matrices which not only adapts well with the biologics of the repair site but also enhances the healing process via providing a cartilage-like matrix [41]. The generation of new tissue with this method is promoted by implantation of cells into a 3-D matrix. The most commonly utilized scaffolds are hyaluronic acid (HA) and collagen-based materials [42,43]. Further, several natural, synthetic and combined materials have been described which can stimulate the mechanical macro-environment of the joint tissue and handle their specifications as commercial products. However, the outputs of clinical studies regarding the application of synthetic multiphase polymer scaffolds have not been adequate in this context [44].

## 4. Nanomaterials for Cartilage Tissue Engineering

The application of novel methodologies in nanotechnology such as 3-D fiber deposition and electro-spinning has led to the enhancement of nanoscaffold quality [45]. Also, the integration of nanoparticles into biomaterials has been shown as a beneficial technique that mimics the ECM and provides the interaction of the cells with the scaffolds, resulting in more functionalized tissue engineering constructs [46]. In addition, cellular behavior is highly connected with nano-features that might affect cytoskeleton, focal adhesions, cell morphology and the expression of several genes such as integrin proteins [47]. Extensive physiochemical methods have been applied for the creation of geometrically relevant nanostructures in the synthesis of scaffolds [48]. Nanofibers, which are mainly constructed with electro-spinning technologies, have been extensively applied in cartilage tissue engineering [49]. The other useful nanoscale materials are nanocomposites. Different polymer-hydroxyapatite nanocomposites are mainly developed for bone repair which also hold promise in cartilage regeneration [50]. Moreover, the biological, electronic and optical superiorities of metallic nanoparticles have made them favorable tools in cartilage repair [51].

## 5. Biomaterials for Cartilage Tissue Engineering

According to reports, a biomaterial can be defined as any nondrug substance with the ability to use in treating or replacing any tissue/organ [52]. This description obviously defined biomaterial in relation to drugs. Therefore, a clarification is needed for this definition of the impression that natural products are synonymous with drugs [53]. We assumed the following description for biomaterials in this study: a biomaterial is any substance (other than a drug) or combination of substances (synthetic or natural in origin) that can be utilized any time, as a whole or as a part of a system for treating, augmenting or replacing any tissue, organ or function of the body [54].

Different research fields such as material sciences, molecular biology and nanotechnology are combined in order to have optimal articular tissue engineering [55]. Also, a strategy which mimics the original niche of the cartilage tissue using a combination of cells, scaffolds and signaling molecules are needed [56]. A 3-D construction and the use of computed tomography (CT) systems will aid researchers in this context [57]. Different biomaterials could be applied (Table 1), which will be discussed below.

## 6. Natural Materials

Natural materials, which are generally employed in effective and satisfactory cartilage repair, include polysaccharide-based (chitosan, alginate, agarose and hyaluronan) and protein-dependent scaffolds (fibrin and collagen) [7,75]. Cell migration, extracellular molecule production, and proliferation occur through the interaction of these natural materials with specific surface receptors [7]. The aforementioned scaffolds will be explained in the following paragraphs concisely.

### 6.1. Agarose

Agarose is a linear polysaccharide of restating units of l- and d-galactose, which has been commonly utilized as a matrix in cartilage tissue engineering. Numerous evidences suggest that this compound is appropriate for the encapsulation of chondrocytes in particular. In addition to their entrapping role, generation of chondrocyte-derived glycosaminoglycan (GAG) highlights their beneficial application in cartilage research [7,75]. In this regard, Awad et al. demonstrated the production of proteoglycan, hydroxyproline, and sulfated GAG (sGAG) in differentiated adipose-derived adult stem cells to the chondrocytes, in the presence of transforming growth factor beta 1 (TGFβ-1), which were seeded in alginate hydrogels and agarose [76]. Additionally, Mouw et al. stated the agarose scaffolds showed the most similarity to the native articular cartilage in comparison with other used scaffolds [77]. Moutos et al. demonstrated the similar mechanical properties of a cell-agarose hydrogel scaffold to the native articular cartilage [78]. A study performed by Tan et al. demonstrated that immature bovine articular chondrocytes entrapped in agarose hydrogel exert a reparative capacity, unlike native cartilage. The engineered cartilage could be repaired in culture provided that the bulk integrity of the developing tissue remains intact [79].

### 6.2. Alginate

Alginate, a natural poly-anionic polysaccharide that forms biodegradable scaffolds, has been extensively used in tissue engineering as a cartilage substitute. Owing to its biocompatibility (in ultrapure preparation technique) and gelling properties, alginate could support chondrocyte phenotype. Similar to agarose, alginate facilitates cell proliferation and migration and the production of extracellular molecules through specific surface receptors [7,75].

Commercial alginates are derived from brown seaweed (Phaeophyceae) and applied as thickeners, gelling agents and stabilizers, and in the food and drug industries. The building blocks of alginate include α-l-guluronic acid (G) and β-d-mannuronic acid (M). The species of the plant and the used segments determine the M/G ratio. The synthetize of alginate commences at fructose-6phosphate as a chief block that is converted to G and D parts intra-cellularly. The M/G ratio not only affects the physiochemical properties of alginate but also result in the generation of different alginate types [80].

Up until now, many studies have been performed to demonstrate alginates efficacy as a natural scaffold. For instance, Cohen et al. showed chondral and osteochondral defect repair by exploiting alginate hydrogels [81]. Moreover, Scholten et al. used the combination of alginate microspheres with a porous polyvinyl alcohol hydrogel scaffold and demonstrated the feasibility of the scaffolds replacement in cartilage defects owing to their role in managing mechanical specifications and enhancing cellular migration [82]. It has been shown that seeded articular chondrocytes in alginate hydrogels increases of the Young’s modulus over time while the mechanical stiffness extended the possessions of initial hyaline cartilage [83]. Mata et al. reported the chondrogenic capability of human dental pulp stem cells (hDPSCs) seeded in alginate hydrogel to regenerate cartilage [84]. Filardo et al. studied the chondrogenic potential of biphasic alginate scaffold in rabbit and sheep models. The implantation of the scaffold provided partial osteochondral regeneration without apparent inflammatory reactions in the animal model [85].

### 6.3. Chitosan

Chitosan, a linear cationic copolymer of N-acetylglucosamine monomers and glucosamine, is a naturally derived polysaccharide that consists of deacetylated chitins. Due to containing GAG, HA, and other similarities to the ECM of cartilaginous tissues, chitosan resembles native cartilage and has been acknowledged in the realm of cartilage tissue engineering. Other advantages of this natural polysaccharide include bioactivity, biocompatibility, biodegradability, anti-bacterial, non-antigenicity, non-immunogenicity, as well as non-cytotoxicity. Further, its beneficial characteristics, i.e., lack of fast gelling properties, augment concerns of forming ectopic cartilage-like tissue in joints [7,75,86]. In this regard, Hoemann et al. introduced a cyto-compatible chitosan solution which showed fast gelling properties (within minutes) and provided accumulation of cartilage matrix by primary chondrocyte both in vitro and in vivo. The gel could reside in a full-thickness chondral defect at least one day and in a mobile osteochondral defect for at least one week [87]. Further, in another study, injectable chitosan-pluronic (CP) hydrogel was applied, in which the proliferation of chondrocytes and GAG production made the new scaffold system a promising tool in the field of cartilage regeneration [88]. Nowadays, describing new chitosan-based scaffolds gain much attention. Different solution of chitosan/poly (3-caprolactone) (PCL) is considered as a new scaffold in this context. The scaffolds containing 75 wt% chitosan and 25 wt% PCL showed the largest neo-cartilage formation while the mechanical specifications of these new scaffolds which contain 50 wt% PCL were higher [7]. Hao et al. reported the reparative and reconstructive role of cell-seeded temperature-responsive chitosan hydrogels in articular cartilage defects in vivo which made them a good candidate in cartilage tissue engineering [89]. Moreover, the beneficial impact (ECM accumulation and collagen type II production) of chondrogenic differentiation of hMSCs that seeded onto chitosan-poly (butylene terephthalate adipate) mesh scaffolds has been reported [90]. Abarrategi et al. studied deacetylation level, molecular weight, and calcium amount within osteochondral scaffolds in vivo and stated that chitosan scaffolds with lower deacetlyation level and molecular weight which have a calcium content of about 18 wt% show optimal results [91]. Evidence suggested the promising role of chitosan/blood implants in comparison to the chitosan free implants. Chitosan fills the defect thoroughly via stabilizing blood clots and the inhibition of its shrinkage. Further, a higher number of hMSCs and an improvement of mechanical properties were seen in chitosan scaffolds [7].

### 6.4. Hyaluronan

Hyaluronan is a highly hydrated, polyanionic, non-sulfated GAG molecule and the ubiquitous element of cartilage ECM. Hyaluronan has gained great promise in the field of tissue engineering and natural scaffolds due to its consistency, biocompatibility (in high molecular weight), viscoelasticity, limited immunogenicity, hydrophilic properties, living cells entrapping capacity, proliferation, and differentiation [7,75]. Lisignoli et al. verified the ability of a commercial hyaloronan-based polymer (Hyaff-11), in supporting hMSCs chondrogenesis differentiation. They reported the upregulation of collagen type II, IX, and aggrecan, as well as the declined expression of collagen type I. Further, they showed that hMSCs differentiation toward chondrocytes was induced in the presence of higher concentrations of TGFβ-1 [92]. Polysaccharides derived from micro-organism sources have multiple industrial applications [93,94,95]. The advantages of this biomaterial include water-solubility, gelling capacity by lowering the temperature, and biological properties (such as non-cytotoxicity and cyto-compatibility) which make them suitable candidate for cartilage tissue engineering [96]. Neethu et al. investigated the potential of chitosan-hyaluronic acid dialdehyde hydrogels in vivo in this context. The results showed that the hydrogels are suitable for bone marrow cells to differentiate, further liberating a combination of hyaline and fibrous extra cellular matrix [97].

### 6.5. Collagen

Collagen as a natural protein is the most insoluble fibrous component present in ECM, which serves as a scaffold substitutes with good cell adhesion properties [7]. In spite of their beneficial effects, these scaffolds have poor mechanical strength that restricts their applications. However, this issue can be addressed by physiochemical modifications such as using cross-linking materials and its combinations with other polymers [75]. Mueller-Rath and coworkers developed a stabilized type I collagen hydrogel seeded with human articular chondrocytes that could migrate and generate ECM proteins [98]. The mechanical specifications of different collagen scaffolds have been evaluated. It has been shown that the modified collagen scaffolds showed increased elastic moduli, nevertheless, their mechanical properties did not reach the properties of native cartilage [99]. Another study in a rabbit model was done by Chen et al. to evaluate the beneficial role of type II collagen scaffold as a substitute for cartilage defects. They reported the production of chondrocyte-like cells with lacuna structure and extracellular molecules without signs of inflammation after eight weeks [100]. Kon and coworkers exploited a novel collagen-based biomaterial (collagen/hydroxyapatite) as a substitute for replacement of cartilage and subchondral bone in the clinic for up to two years. They reported promising results, especially in young and active patients [101]. Zhang et al. reported the chondro-inductive capability of type I collagen hydrogel seeded with rabbit bone marrow MSCs (BMSCs) without exogenous growth factors in vitro [102]. Miao et al. investigated the proliferation ability and phenotype preservation of different hydrogels and they reported that the collagen scaffold can support chondrocyte proliferation and conserve the cell phenotype better than other hydrogels in the study [103].

### 6.6. Fibrin

Fibrin, a blood protein, is involved in the natural blood clotting process. Human fibrin gels which are FDA-approved material, mimics clotting process and could be used as a matrix in cartilage tissue engineering. The fibrin hydrogel stimulates GAG production and formation of the ECM in fibrin-chondrocyte co-culture [7]. Peretti et al. reviewed the studies of fibrin hydrogels in articular cartilage repair in laboratory animals and suggested that the mixture of autologous chondrocytes and allogenic devitalized cartilage matrices suspended in fibrin glue led to the generation of cartilage-resembling constructs [104]. In another study, a research was conducted on cases with extensive articular cartilage damages. Their BMSCs were seeded into platelet-rich fibrin glue (PR-FG). After a year of follow-up, three out of five patients exhibited a comprehensive defect recovery, in comparison with the other two, which showed inadequate harmony with original cartilage [105]. Rampichová et al. demonstrated the efficacy of fibrin/HA composite hydrogel scaffold in pig knee cartilage regeneration [106]. Iseki et al. investigated the efficacy of fibrin gel scaffolds encapsulating MSCs and mechanical load in the generation of hyaline-resembled cartilage repair tissue [107]. Further, Krug et al. reported that fibrin glue acts as an appropriate biomaterial for delivering adipose-derived multipotent stem/progenitor cells (ASPCs) to the damaged tissues [108].

## 7. Synthetic Materials

Besides natural materials, synthetic compounds represent an interesting topic for cartilage research. Different types of conventional materials are used in this field namely poly polylactide acid (PLA), (N-isopropylacrylamide) (NiPAAm) and derivatives (PLLA, PLGA, PDLA), polyurethane (PU), poly(ethylene glycol) (PEG), and poly (vinyl alcohol) (PVA). They provide good potential for processing in addition to excellent mechanical properties (Young modulus of native cartilage range from 0.2 to 0.3 GPa) [109]. Hydrogels show high efficiency to entrap a wide array of live cells as well as creating a highly hydrated niche, enabling easy diffusion of nutrients and inducing cellular migration, proliferation and differentiation [110]. Poly(ethylene glycol) (PEG) has widely been studied as a supporting agent in cartilage tissue engineering. In rigid or hydrogel scaffold types, it has been tested to promote the cellular viability, attachment, growth, and ECM generation of seeded chondrocytes [111].

In spite of the feasibility of PEG in cartilage tissue engineering alone, various researches have investigated the compression modulus and high strength when mixed with other natural and synthetic materials [112,113]. Rakovsky’s group [112] prepared PEG hydrogels and amphiphilic interpenetrating polymer networks (IPNs) of PEG with poly methyl I methacrylate (PMMA) and characterized their molecular weights, PMMA volume fraction, and cross-link densities. The authors reported that lower molecular weight, increased values of cross-link concentration and higher PMMA levels result in lower water content and higher equilibrium modulus. However, IPNs increased the strength of the hydrogel, making them ideal materials for use as cartilage replacements. The effect of an injectable PEG-albumin hydrogel supplemented with HA was monitored by Scholz et al. [113]. Vascularization is not observed in native articular cartilage, while cartilage degradation increases after the formation of blood vessels in disease conditions. For this, subcutaneous implantation of PEG-albumin hydrogel seeded human chondrocytes was performed in immunodeficient mice. The results showed no vascularization with the scaffold after 14 days. Also, the preservation of specific genotype expressing type I and II collagen and aggrecan by chondrocytes, proved that these scaffolds are promising implant in cartilage engineering. Liu and co-workers [114] developed a collagen mimetic peptide (CMP) comprising a GFOGER (collagen-mimicking action) sequence flanked by repeats of GPO ((GPO) 4 GFOGER (GPO) 4 GCG, CMP) incorporated into a PEG hydrogel as a novel strategy in the differentiation of hMSCs into cartilage–like tissue. The histopathologic results demonstrated an increase in the accumulation of type II collagen and aggrecan in cells present in the hydrogel matrix. Further, activation of cartilage specific genes and increased accumulation of ECM were detected. Poly(N-isopropylacrylamide) (PNiPAAm) is a temperature-sensitive polymer consisted of polyacrylic acid, which undergoes a reversible lower critical solution temperature (LCST) phase transition at 32 °C [115]. When this agent is copolymerized with acrylic acid, a gel is produced at 37 °C that can turn into liquid form at lower temperatures. A similar scaffold was developed and test in a rabbit model of cartilage defect. Both vascularization and inflammatory response were missing five weeks after implantation. The results of this study indicated superior properties of PNiPAAm hydrogels to support cartilaginous tissue reconstruction with no leakage of the transplant or surface deformation. Ibusuki’s group [116] prepared and evaluated an injectable gellable PNiPAAm-grafted gelatin scaffold embedded with chondrocytes. Mechanical properties and total collagen and s-GAG extended to native cartilage standards at the end of 12 weeks. Moreover, in another study an injectable chitosan-PNiPAAm hydrogel was developed [117] with an LCST around 30 °C. According to the results, chondrocytes entrapped within hydrogel preserved their vitality and phenotypic morphology.

More importantly, lower NH_2_/COOH ratio copolymers exhibit a strong mechanical strength and fast sol–gel transition compared to the PNiPAAm hydrogels, thus being perfect scaffold candidates. Park et al. [118] synthesized PNiPAAm-co-vinylimidazole-p(NiPAAmco-VI)-based hydrogel containing chondrocytes and growth factor containing NPs for cartilage regeneration, providing a suitable model for maintaining the cell phenotype.

PLA and its related polymers (poly (Dlactide) (PDLA), poly (l-lactide) (PLLA) and poly (lactic-co-glycolic acid) (PLGA), have been reported as a scaffold to promote cell proliferation and differentiation in cartilage tissue engineering research [119]. Recently, Tanaka et al. [120] tested different scaffolds with various ranges of pore sizes and porosities, consisting of polylactide and correlated polymers. The prepared scaffolds embedded with a mixture of chondrocyte/atelocollagen and implanted subcutaneously in nude mice. The macroscopic and histological analyses demonstrated that the 3-D shape of scaffolds was preserved two months after implantation. Further, a significantly larger amount of both collagen types and GAG were found on PLGA and PLLA scaffold used in implantations. Moreover, quantitative analysis of total macrophage numbers surrounding the scaffolds demonstrated excellent results for PLGA and PLLA. In fact, lesser numbers of macrophages were detected within these scaffolds in comparison with the resting ones, proposing their higher affinity as cartilage surrogates. For increasing cell attachment and proliferation, a bioactive atelocollagen-grafted PLLA membrane was examined and subjected to DC-pulsed oxygen plasma treatment [120]. Positive cell vitality, proliferation, and differentiation rates were shown to be positive for chondrocytes seeded into this membrane. This finding was later supported by the liberation of GAG and collagen. Further, chondrocyte morphology and structure were maintained throughout the sampling period, demonstrating their potential for this issue. This team also made a suggestion to improve the quality of their earlier research, linking cationized gelatin with PLLA membranes, which showed an upregulation of specific markers such as type II collagen, aggrecan, and SOX-9 [121]. Histology and immunostaining showed that this membrane was capable of forming ectopic cartilage at 28 days after subcutaneous implantation. In separate studies, some researchers evaluated the mechanical features of PLA derivatives. Zhao and coworkers [122] introduced a porous microstructure with a compressive modulus in PLLA scaffolds. In another study, nanofiber-based PLGA scaffolds with various ratios of glycolic acid/lactic acid were examined by Shin et al. [123]. Here, final tensile stress, tensile modulus, and resultant strain values reached maximum values to the cartilage in an adult human. Furthermore, cell proliferation and ECM deposition were enhanced in these scaffolds types.

The use of polyurethanes as articular cartilage substitutes offers a number of potential advantages such as ease of processing in the form of an injectable gels/paste, the possibility of in situ polymerization, and good mechanical properties [124]. A recent attempt was made to develop porous polyurethane systems compromising zwitterionic constituents dihydroxypolycaprolactone phosphorylcholine (DPCLPC), and 1,2-dihydroxy-N,Ndimethylamino-propane sulfonate (DAPS) [125]. Scaffolds were also prepared using polymers combined with hydrated gelatin beads, conferring an acceptable compression. A moderated degradation of polymers was observed after two months following in vivo implantation and increased time-dependently. Meanwhile, DPCLPC-consisting polymers are desired to better deliver chondrocytes and growth factors. The effect of urethane-based scaffold composed of polymer segments of monohydroxy dimethacrylate poly (ε-caprolactone) triol and diisocyanato poly(ethylene glycol) was investigated by the same group [126]. Based on the results, a micro-sized capsule generation and slight host tissue response were observed after in vivo implantation. The cells were cultured in vitro onto these scaffolds and maintained for up to eight weeks in static and dynamic conditions, promoting cell proliferation, migration, and ECM production. These cells could induce the expression of collagen type II and IV and keratin sulfate similar to native articular cartilage. In another study, Grad et al. [127] examined cell-seeded porous polyurethane scaffolds but they exhibited less resembles properties to native cartilage tissue.

Polyvinyl alcohol (PVA) is a synthetic water-soluble polymer that shows exceptional adhesive characteristics. Because of its superior performance to catch live cells, PVA shows great promise in cartilage repair. Charlton and co-workers [128] engineered a semi-degradable PVA-PLGA scaffold with comparable mechanical possessions to native cartilage. In another research by Holloway et al., the ability of PVA-based hydrogels reinforced with very high molecular weight polyethylene was examined that showed high tensile modulus, but its use is limited since the scaffold is non-degradability.

Usually, scaffolds with higher contents of PLGA seem to be a more suitable support for articular cartilage substitutes. Notably, a higher PLGA content leads to scaffolds with larger pores, promoting the migration of cells into the scaffold. Bichara et al. [129] analyzed the potential of human nasal septum chondrocytes for neocartilage formation within a PVA-alginate hydrogel.

Histological data demonstrated ample intensities of type II collagen and GAG in comparison with the tissue all over the PVA. Constructs cultured in bioreactors had a significantly larger equilibrium compressive modulus than scaffolds implanted promptly without pre-exposure to a bioreactor, thereby representing a promise to be applied in cartilage tissue engineering.

In addition to scaffolds and cell sources, growth factors can be used for engineering articular cartilage. For this, a scaffold of PVA-poly (caprolactone) seeded with MSCs and variations of growth factors was proposed by Mohan et al. [130]. In this study growth factors could strongly affect stem cells’ shape, differentiation, distribution, and liberation of ECM molecules. They reported that the combination of some growth factors resulted in higher differentiation of stem cells into chondrocytes compared to the others. Although several studies have examined several combinations of scaffolds, cells, and growth factors, Tran et al. [131] investigated a scaffold-free cartilage construct. In another recent study, a large amount of tissue-engineered cartilage was developed from porcine chondrocytes by centrifuging a chondrocyte cell suspension at high-density onto an agarose layer and relocating it into a bioreactor for up to one month. The synthetized scaffolds could be easily manipulated without any attachment to agarose layer in static culture. In contrast to the static culture situations, an ECM rich in proteoglycans was observed as dynamic environments, suggesting the potential application of a bioreactor to promote both the biomechanical and biochemical characteristics of engineered tissue. Over the past years, various natural and synthetic biomaterials have been examined in vivo or during clinical trials for cartilage replacement, but additional studies are needed to define the long-term efficacy of these biomaterials. The most widely studied scaffolds for clinical cartilage regeneration are collagen scaffolds [10,132].

Type I and III collagen scaffolds are commonly applied in matrix-related autologous chondrocyte transplantation. This strategy considerably enhanced postoperative values in comparison with prior results [10]. Hyaluronan-based scaffolds are of interest for cartilage reconstruction in addition to collagen-based ones [133,134]. A fibrin-based carrier system was used for autologous chondrocyte transplantation by Kim and co-workers [135]. MRI examination and a second arthroscopy were performed 12 and 24 months after implantation. In agreement with the aforementioned investigations, the patients observed improvements in functional and clinical outcomes.

Synthetic materials have also been assessed in clinical trials. A polymer-based scaffold of polyglycolic/polylactic acid polydioxane, BioSeed-C (BioTissue Technologies, Freiburg, Germany), was applied for cartilage defect treatment [136]. Autologous chondrocytes were cultured, expanded, and reorganized in the scaffold. Results revealed substantial progress in pain drop and quality of life over a two-year period in 40 patients. According to the results, these biomaterials had significantly improved final postoperative values, thus representing their effectiveness in cartilage repair. Autologous chondrocytes were cultured during a first surgery, prior implantation in a 3-D matrix. In the next step, the transplantation of cell-scaffold constituents was performed. For this, novel treatment methods are developed, which can make the patient more relaxed.

## 8. The Next Generation Biomaterials for Cartilage Tissue Engineering

Agarose, alginate, hyaluronan, collagen type I gels and sponges, collagen type II sponges, PLA, PGA, and fibrin are considered as classical carriers in articular chondrocytes [137]. The advances in matrix incorporation and alterations in equilibrium modulus can differ based on the type of materials. The development of nano-biomaterials for cartilage tissue engineering applications needs a fundamental understanding of the interactions between polymer sciences, nanotechnology field, and cell biology. This combination suggests the best models/approaches for the development of high-tech nano-biomaterials with the possession of cartilage.

Hydrogels are one of the smart materials in cartilage scaffolds due to their innate hydrated structure, distinctive biocompatibility and capacity to join chemical cues [138]. These benefits promote the development of ECM-like matrices derived from molecular building-blocks, such as elastin-like polymers [139], peptide amphiphiles [140] or the commercially available Puramatrix (3DM) [141], that imitate the function and structure of native ECM. Empirical works in animal models of osteoarthritis have shown the beneficial impact of these constructs [142]. According to the gathered data, constructs containing matrices with cells were more valuable than matrices alone in treating defects [142]. Due to these benefits, generating treatment strategies using both constituents for articular cartilage regeneration/repair is more useable. Hydrogels based on PEG macromers, particularly diacrylate forms, are able to be gelled into multifaceted defects. This ability made them to be considered during years ago [143]. In 2007, a specific PEG hydrogel was developed that evoke the capacity to use as a platform to exhibit specific biomolecular signals [144]. This PEG hydrogel was able to enhance cell attachment and degradation selectively [144]. In 2011, the complexity of these PEG-associated networks was increased when chondroitin sulfate and matrix metalloproteinase sensitive peptides were incorporated [145]. Since then, a huge number of different diversities of PEG hydrogels with a capacity of use in cartilage repair have been fabricated [145,146]. A diversity of hydrogels with hyaluronan has been introduced for hyaline cartilage regeneration. This complex could mimic the natural tissue properties and provide a highly hydrated environment. However, their mechanical strength was low and further modifications were required to improve their handling [147]. Some modifications like use of chemical cross-linking compounds may cause toxicity. Researchers have developed different strategies in order to enhance the functionality of HA-related hydrogels. One approach has been the design of composite hydrogels, whereby structural proteins are incorporated within the HA material, such as the development of fibrin within HA. Fibrin/HA hydrogels display promoted mechanical specifications, improved ECM formation, and have the capability to deliver cells [148]. Another example of composite hydrogels is the incorporation of collagen type I within the HA. In this incorporation, chondrocyte growth and proteoglycan synthesis are stimulated, and mechanical properties improved. Although HA molecules show several superiorities, their impact on the mechanical specifications of a hydrogel, degradation properties, and the capability to preserve and release growth factors have been described as the most investigated properties to be used in cartilage regeneration [149,150]. For instance, the modification of HA with methacrylate has provided the platform for UV-based polymerization of the hydrogel [151,152]. Also, the application of these materials has improved the encapsulation of chondrocytes and stem cells [150].

Thermo-sensitive HA scaffolds are valuable innovations that have been introduced recently. These platforms are corporations of a modified HA compound and thermo-sensitive PNIPAM parts [153]. Above a specific temperature, the conformation and assembly of these modified HA chains will change and promote the formation of stronger hydrogels that support cell survival. This self-thermo-sensitive-assembling invention provides injectable HA platforms that could be inserted in the site of injury through minimally invasive procedures. At body temperature, the modified HA chains will assemble to the stronger hydrogel.

Nanohydrogel benefits from the advantages of hydrogel and nanoparticle-based systems. Many studies have focused on preparing nanohydrogels to alter the physical and chemical properties of gels. Nanogels have some improved properties in comparison to gels, such as an improved permeability, better environmental response, better surface function, improved biodegradability, as well as better bio-recognition, which qualifies them for a special biomedical application like tissue engineering [154]. Several self-assembling materials based on small molecular building-blocks are being fabricated [155]. The other form of self-assembling materials is short β-sheet forming peptides. These scaffolds are capable of self-assembling to a different form of nano-scale constructs such as fibrils, ribbons, tapes and shape gels with adjustable physiochemical characteristics [156]. β-hairpin peptides are the other form of self-assembling materials that bend intramolecularly and achieve an amphiphilic structure, an important construction to form self-assembled hydrogel networks [157]. Furthermore, molecular self-assembling platforms are promising innovations to improve the general future of hydrogels in mimicking fibrocartilage properties. Collagen-mimetic self-assembling peptides are one of the most worth full examples of these systems [158]. As an attractive property, these systems provide hydrogels with a high water content and structural integrity near to fibrocartilage. Repeating positive and negative l-amino acids that self-assemble into nanofibres through β-sheet interactions, are the other design of nonafibre scaffolds [159]. This material has been used to stimulate the growth of chondrocytes, and the fabrication of main hyaline cartilage markers by Liu et al. [160]. The other promising self-assembling scaffolds have been introduced by Semino et al., that used a self-assembling peptide scaffold RAD16-I (Puramatrix) to produce cartilage tissue from mouse embryonic [161]. The co-assembly network of peptide amphiphiles (PAs) shows a high density of binding epitopes for TGF-β-1 [162]. Shah et al. showed the viability and differentiation of hMSCs to chondrogenic cells [162]. Furthermore, they proved the repair of a rabbit model. Also, electrospun nanofibres are the other platforms that facilitate the adhesion of chondrocytes and maintain their phenotype. In this innovative design, the polymer solution receives a high electrical potential [163]. One example of electrospinning is 3D nanofibrous PCL scaffold consisting of electrospun nanofibres capable to support the matrix deposition and cellular phenotype [164]. PCL and polylactic acid microfibres scaffolds with high porosity [165] and degradable copoly(ether) esterurethane (PDC) or poly(p-dioxanone) (PPDO) using 1,1,1,3,3,3 hexafluoro-2-propanol (HFP) as a solvent are the other design of platforms in recent years [166]. Degradable polymers are good candidates for biomaterial-dependent cartilage regeneration, especially where the diameter of the fibers are nanometer-size ranges [167]. It has been proven that the growth of chondrocytes in such nanofibrous platforms is acceptable and this type of culture preserves the capability of chondrocytes in the production of cartilage markers. By the application of PCL scaffolds, the nanofibrous scaffolds are well tolerable and can encourage the development of tissue repair by the co-insertion of both MSCs and chondrocytes [168].

**Figure 1:**
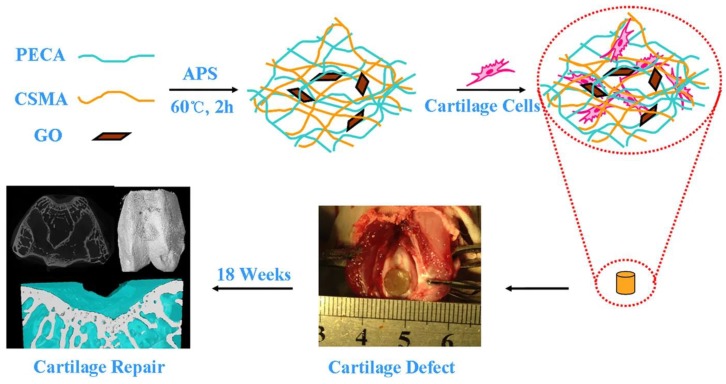
CSMA/PECA/GO hybrid scaffold for cartilage regeneration. Adopted from [169], under the terms and conditions of the Creative Commons Attribution (CC BY) license.

Liao et al. designed a hybrid scaffold composed of graphene oxide (GO) nanoparticles, methacrylated chondroitin sulfate (CSMA), and poly(ethylene glycol) methyl ether-ε-caprolactone-acryloyl chloride (PECA) for application in cartilage regeneration. The scaffold showed a suitable porosity, pore size, swelling capability, conductivity, and compression modulus to imitate the natural ECM of cartilage. In the case of cartilage tissue engineering, GO nanoparticles could be considered as a talented candidate. This may improve the topographical, mechanical, and electrical cues in the scaffold matrix. CSMA/PECA/GO scaffold containing cells showed improved chondrocyte shape, integration, incessant subchondral bone, and much thicker cartilage-like tissue in comparison with scaffold other groups (Figure 1) [169]. In another work, incorporating GO nanoparticles into a scaffold leads to the formation of a self-healing nanocomposite scaffold with good mechanical strength [170].

The generation of zonally-distinct cartilage layers on nanofibers, the inclusion of sacrificial fibers, and the application of ECM proteins in order to prepare nanofibers have been listed as recent advances in cartilage regeneration [171] which will open a new avenue in this context.

**Figure 2:**
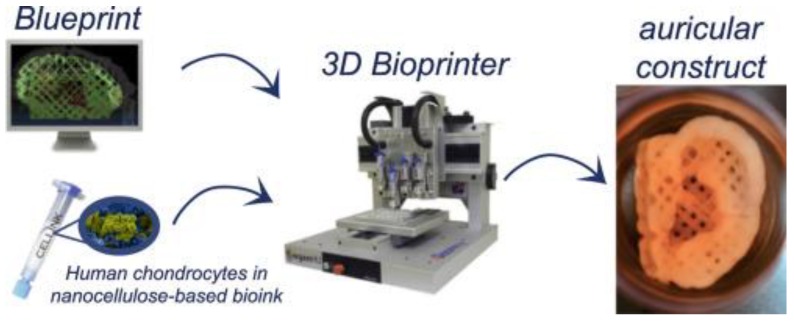
3D bioprinting process of chondrocyte-laden NFC-A auricular constructs with open porosity. Adopted from [172] with permission.

Bioprinting technology has achieved attention in numerous tissue engineering approaches for its capability to spatially regulate the location of materials, cells and other biological molecules. Though significant developments have been seen in bioprinted complex 3D tissue analogues, the production of hydrogel bioinks with great printability and bioactive specifications should be promoted in order to have an acceptable clinical use. Ávila et al. assessed the biological efficacy of a bioink composed of nanofibrillated cellulose and alginate (NFC-A) for auricular cartilage tissue engineering which facilitated the biofabrication of the cell-laden, patient-specific auricular construct, high cell density and equal cell spreading. NFC-A bioink supports the neo-synthesis of cartilage-specific ECM components and the redifferentiation of hNCs. In addition, the cell-laden NFC-A constructs showed a great shape and size stability as well as an enhancement in proliferation and viability of cells. This confirmed that NFC-A bioink offering suitable printability in a biologically appropriate aqueous 3D environment, turning it to a proper strategy for auricular cartilage tissue engineering (Figure 2) [172].

## 9. Clinically Use of New Tissue-Engineered Approaches

Articular cartilage, as a load-bearing tissue, regulates the biomechanical properties of the body via a variety of mechanical and biological properties [173]. However, it is very susceptible to injury and degenerative disease in different parts of the body [174]. The clinical approach for managing these discomfort conditions is related to the patient’s age, anatomical location of the joint harboring the articular cartilage defects, and the dimension and depth of the lesion [175]. Furthermore, the use of tissue-engineered methods is considered as one of the newest approaches. Whether the defect is osteochondral or purely chondral in nature and in relation to the form and degree of the injury, the type of algorithm in this approach varies. Several biomaterial scaffolds with therapeutic effects against cartilage disease or trauma are present in the market (with many more in the pipeline) [176]. These biological platforms provide an appropriate matrix for regulating the fate of implanted cells. Hyaluronan and collagen-based biomaterials are considered as the most popular biomaterial for articular cartilage healing. However, the impact of the materials on the cell survival and the type of tissue shaped has only been briefly investigated [177].

The most prominent challenge in the use of stem cells for differentiation into the chondrocyte phenotype is the avoidance hypertrophy, which demands biological, chemical and physical regulations [178]. The translation of these biomaterials into the patient’s bed is the most rate-limiting aspect of this type of study. Evaluating the safety of these materials individually is one of the first important aspects, requiring time and economical investment. Recently, a poly(ethylene glycol) diacrylate (PEGDA) hydrogel in combination with microfracture has been used in focal defects of 15 patients [179].

## 10. Conclusions and Future Outlooks

Over the last decades, the researchers have developed newly formed articular cartilage replacements. They have attained a worthy comprehension of the physical, chemical, and morphological properties of native articular cartilage. Moreover, they made forward investigations, specifically in the application of cutting-edge printing techniques for imitating articular cartilage tissue. Nonetheless, the investigations should be extended to an accessible, simple and cost-effective approach to the treatment of articular cartilage damage, otherwise, surgeons may follow the traditional approaches for the treatment of patients with chondral defects. Despite developments in the design of scaffolds and bioreactor situations, engineered cartilage constructs remain mechanically inferior to native cartilaginous tissues. Furthermore, the engineered tissues displayed far weaker integration and quality deterioration over time when assessed in laboratory animals.

Cartilage tissue engineering needs cells, signaling molecules, and an appropriate scaffold to prepare a new cartilage tissue under special settings. The quick development in material science and engineering has caused progress in the alternative medical approaches for different diseases such as cartilage defects. Nevertheless, cartilage tissue engineering is still in the developing stage. A lot of potential variables in this approach existing and the significant problems remain to be solved. Numerous fields including material science, biology and nuclear transfer are involved in cartilage tissue engineering and expert staffs in methods of cell treatment and polymer synthesis are crucial for the effective application of mentioned methods to produce novel cartilage. The forthcoming investigation in this field should be designed to study and assess the application of tissue engineering technologies as well as surgical methods for cartilage repair in diseased animal models to achieve an improved comprehension of clinically viable design. The progress of a model system for the the investigation of cartilage tissue pathologies is vital.

The development of innovative nanomaterials with preferred characterizations could enhance the chondrogenesis of appropriate tissue grafts for the regeneration and repair of cartilage. Recently, some studies performed cartilage tissue engineering, such as nanofibrous scaffolds produced through thermally induced phase separation or electrospinning method, nanosurfaces produced via lithography or acid/base treatments, and nanocomposites produced by integrating of nanomaterials in scaffolds. Nanomaterials lead to produce scaffolds that more closely imitate the natural cartilage tissue in ECM structure. The mentioned process enhances the interaction between the cells, scaffold, and tissue growth. These nanoscale features have displayed achievement in improving cell growth, morphology, differentiation, adhesion, and the production of cartilaginous ECM. An upcoming study in this area should ensure the production of nanocomposites with both biomechanical and biochemical characteristics that imitate the functional and morphological properties of the human cartilage tissue. The produced engineered cartilaginous tissues would be appropriate for final clinical application.

## Figures and Tables

**Table 1:** List of selected biomaterials in cartilage regeneration including their advantages and disadvantages.

	Preparation Source	Advantages	Disadvantages	Ref.
**Natural materials**				
Agarose	Highly purified polysaccharide derived from agar	High water absorbance capacity which is beneficial for cell growth, differentiation and proliferation. Similarity to the ECM which supports cell adhesion with chemical modifications.	Non-degradability because of the absence of suitable enzyme in the body. The addition of agarose had no destructive effect on cartilage tissue and no changes were detected on collagen and DNA content.	[58]
Collagen	Decellularization and demineralization of tissues	Biocompatible, reservoir for growth factor delivery in the ECM. Collagen improve the spontaneous repair process of osteochondral defects in vivo.	Immunoreactivity related to its bovine source and other non-human species.	[59]
Chitosan	Deacetylation of chitin	Biocompatibility, biodegradability, low toxicity, and controlled degradation by enzymes such as lysozyme. Chitosan is capable to improve repair of cartilage, promote chondrogenic activity of chondrocytes and synthesis of ECM proteins in vitro. Chitosan-based matrices stimulate the formation of a hyaline-like repair tissue in articular cartilage defects In vivo.	Poor solubility in neutral aqueous solutions and organic solvents because of the presence of amino groups and its high crystallinity.	[60,61]
Alginate	Brown algae	Biocompatibility and non-immunogenicity. Alginate allows the maintenance of the chondrocytic phenotype and the synthesis of ECM proteins.	Poor cell adhesion, low mechanical strength, and low degradability. Alginate alone prevents spontaneous repair in vivo and when associated with chondrocytes, it did not repair osteochondral defects partly because of severe immunological reactions.	[62,63]
Hyaluronic acid	Rooster cockscomb or from microbial fermentation.	Good bioactivity, biodegradability, biocompatibility, and act as a reservoir of growth factors with chemically modified. Hyaluronic acid based matrices enhance the synthesis of ECM by chondrocytes in vitro and in vivo.	Structural complexity, possibility of immunoreactivity. Hyaluronic acid is degraded naturally by hyaluronidases but its products of degradation are capable to stimulate chondrolysis. Under an unmodified form, HA is not appropriate for cartilage repair and requests crosslinking to improve its biocompatibility.	[64,65]
Fibrin Glue	Polymerization of fibrinogen in the attendance of thrombin.	It stimulates the spontaneous repair action of articular cartilage but also has a pro-inflammatory effect. Fibrin induces its own degradation by the components of ECM into nontoxic endpoint components. The utilizing of fibrin glue and chondrocytes improve the repair of cartilage in vivo.	Low mechanical strength and less controllable biodegradability. In human, its application is limited to seal off the periosteal flap in the ACI method.	[66,67,68]
**Synthetic materials**				
PEG	Chemical synthesis	Non-immunogenicity, good biocompatibility, Low toxicity, great hydrophilicity and solubility in organic solvents, and anti-fouling property.	Non-biodegradable	[69]
PLA	Hydrolysis, or specific cleavage of oligopeptides	High mechanical strength	It stimulate immunological reactions partially and it is cytotoxic.	[70]
PGA	Hydrolysis, or specific cleavage of oligopeptides	High strength and modulus	It stimulate immunological reactions partially and it is cytotoxic.	[71,72]
PLGA	Chemical synthesis	Biocompatibility, and biodegradability rate. In vivo studies displayed the improve cartilage regeneration with application of PLGA caffolds and microspheres with and without loaded drugs.	Expensive and weak cell adhesive ability.	[72]
PCL	Chemical synthesis	PCL can maintain phenotype and promote chondrocytes proliferation. It has slow degradation rate and high drug permeability.	poor hydrophilicity and acidic degradation products which may cause inflammation.	[73]
PNIPAM	Chemical synthesis	It is thermoresponsive polymer which is very important because of its well defined structure and property specially its temperature response is closed to human body and can be finetuned as well.	Non-biodegradable and its monomer and cross-linker may lead to toxic, teratogenic and carcinogenic effects.	[74]
